# GFI-YOLOv8: Sika Deer Posture Recognition Target Detection Method Based on YOLOv8

**DOI:** 10.3390/ani14182640

**Published:** 2024-09-11

**Authors:** He Gong, Jingyi Liu, Zhipeng Li, Hang Zhu, Lan Luo, Haoxu Li, Tianli Hu, Ying Guo, Ye Mu

**Affiliations:** 1 College of Information Technology, Jilin Agricultural University, Changchun 130118, China; gonghe@jlau.edu.cn (H.G.);; 2 Jilin Province Agricultural Internet of Things Technology Collaborative Innovation Center, Changchun 130118, China; 3 Jilin Province Intelligent Environmental Engineering Research Center, Changchun 130118, China; 4 Faculty of Animal Science and Technology, Jilin Agricultural University, Changchun 130118, China

**Keywords:** sika deer, deep learning, object detection, attention mechanism

## Abstract

**Simple Summary:**

Through gesture recognition and detection of sika deer, farmers can observe the gestures of sika deer without physical contact, providing data and technical support for the intelligent and welfare-oriented breeding of sika deer. This study is based on the YOLOv8 network model. By optimizing the convolution module, incorporating the attention mechanism, and enhancing the detection head module, a new method for detecting sika deer poses was developed. The method was assessed using four behavioral datasets, which included standing, lying, eating, and attacking. The pose-recognition accuracy of sika deer significantly improved to an average of 91.6%, laying a foundation for the health assessment and information management of sika deer.

**Abstract:**

As the sika deer breeding industry flourishes on a large scale, accurately assessing the health of these animals is of paramount importance. Implementing posture recognition through target detection serves as a vital method for monitoring the well-being of sika deer. This approach allows for a more nuanced understanding of their physical condition, ensuring the industry can maintain high standards of animal welfare and productivity. In order to achieve remote monitoring of sika deer without interfering with the natural behavior of the animals, and to enhance animal welfare, this paper proposes a sika deer individual posture recognition detection algorithm GFI-YOLOv8 based on YOLOv8. Firstly, this paper proposes to add the iAFF iterative attention feature fusion module to the C2f of the backbone network module, replace the original SPPF module with AIFI module, and use the attention mechanism to adjust the feature channel adaptively. This aims to enhance granularity, improve the model’s recognition, and enhance understanding of sika deer behavior in complex scenes. Secondly, a novel convolutional neural network module is introduced to improve the efficiency and accuracy of feature extraction, while preserving the model’s depth and diversity. In addition, a new attention mechanism module is proposed to expand the receptive field and simplify the model. Furthermore, a new pyramid network and an optimized detection head module are presented to improve the recognition and interpretation of sika deer postures in intricate environments. The experimental results demonstrate that the model achieves 91.6% accuracy in recognizing the posture of sika deer, with a 6% improvement in accuracy and a 4.6% increase in mAP50 compared to YOLOv8n. Compared to other models in the YOLO series, such as YOLOv5n, YOLOv7-tiny, YOLOv8n, YOLOv8s, YOLOv9, and YOLOv10, this model exhibits higher accuracy, and improved mAP50 and mAP50-95 values. The overall performance is commendable, meeting the requirements for accurate and rapid identification of the posture of sika deer. This model proves beneficial for the precise and real-time monitoring of sika deer posture in complex breeding environments and under all-weather conditions.

## 1. Introduction

Sika deer hold significant economic value in the farming industry, with an increasing demand for their products in both domestic and international markets, resulting in substantial economic benefits for farmers [1]. The advancement of modern technology, particularly in artificial intelligence and computer vision, has led to the emergence of automated posture recognition technology, which presents new opportunities for the behavior analysis and health monitoring of farmed sika deer [2]. This study aims to investigate how these technologies can be utilized to enhance the efficiency and quality of sika deer farming while providing a scientific foundation for effective farming management [3]. The primary principle of sika deer posture detection technology involves training a deep convolutional neural network model to identify sika deer in the input image and output the posture information of sika deer. During the training process, the model necessitates a large number of sika deer images with labeled information as training samples to learn the posture characteristics and patterns of sika deer. By developing and implementing sika deer pose recognition systems, breeders can more effectively monitor the health status and behavior of the deer population, thereby optimizing the breeding environment and management strategies. This approach not only enhances the production efficiency of sika deer farming but also ensures the health and welfare of the deer population, supporting the sustainable development of the industry. Furthermore, the application of this technology can promote advancements in related fields, establishing a foundation for future automation and intelligence in farming practices [4].

Object detection plays a vital role in computer vision. By leveraging the power of deep convolutional neural networks, models can learn complex feature representations from images, enabling them to accurately detect and localize objects. In recent years, deep learning has been widely applied to animal behavior recognition (Li et al., 2020) [5], agricultural product inspection (Deng et al., 2019) [6], and other fields. Simultaneously, some researchers have started using deep learning techniques to study the living habits of animals. For instance, deep learning technology is utilized to detect the farming environment and contour information of cattle. To acquire the health information of cattle, Qiao et al. proposed a Mask R-CNN-based method to address the instance segmentation and contour extraction challenges of cattle in a real farm environment [7]. Wang et al. introduced a YOLOv8-based estrus cow recognition model to tackle the issue of real-time monitoring of cows’ estrus period in a farm environment, enhancing target detection efficiency [8]. To enhance the accuracy of detecting key parts of sika deer, considering the diverse deer farm environment and the swift movement of sika deer, Xiong et al. proposed an AD-YOLOv5 algorithm for key parts detection based on YOLOv5s [9]. Shao et al. employed deep separable convolution to distinguish the standing, lying, and side-lying postures of pigs, achieving an accuracy of 92.45% [10]. To enhance the detection of facial emotions in pigs and analyze their emotional cues, Nie et al. proposed a deep learning strategy based on CreToNeXt YOLOv5 for advanced pig facial emotion detection [11]. Gong et al. proposed the behavior recognition of sika deer [12]. An enhanced Google Inception Network (GoogLeNet) model was utilized for behavior identification. The model achieved a recognition rate of 98.92% for red deer behavior. Wu et al. utilized CNN-LSTM to identify the basic behaviors of individual cows in complex environments, and further enhanced the recognition accuracy of five postures: drinking water, estrus, walking, standing, and lying [13]. Yu et al. conducted a study on automatic recognition of daily cow behaviors using deep learning, enabling the model to accurately identify various behaviors exhibited by cows in real cowshed environments [14]. The rapid advancement of deep learning technology has demonstrated significant potential in image recognition and analysis. However, current target detection models may face limitations in specific environments, such as complex lighting conditions on farms, or regarding the diverse range of animal behaviors. Additionally, in the field of sika deer farming, there is currently no model that is highly suitable for farmers to remotely detect and identify sika deer postures. To enhance the accuracy of sika deer posture detection and recognition, it is essential to refine the existing model to adapt to these unique environments and the requirements of sika deer farming.

In order to solve the aforementioned issues, this paper introduces an enhanced model for sika deer posture recognition based on YOLOv8. This model enhances the fundamental model in various ways, such as introducing an efficient multi-scale attention mechanism, enhancing a novel pyramid network structure model, improving the structure of the original C2f model, and refining the detection head module of the original model. By evaluating the performance using the self-constructed sika deer dataset, the GFI-YOLOv8 model has demonstrated commendable accuracy, recall, and average precision, showcasing notable enhancements. The key contributions of this article are as follows:

(1) In this paper, Iterative Attentive Feature Fusion (iAFF) [15] is introduced into the C2f structure to form the C2f_iAFF. The original Spatial Pyramid Pooling Fast (SPPF) module is replaced with the attention-based intra-scale feature interaction module (AIFI) [16] module. This enhancement aims to improve the model’s performance in object detection and recognition by iteratively fusing features at different scales. It focuses on processing advanced image features through a self-attention mechanism.
(2) This paper proposes a new attention mechanism module, which is combined with the CSP-Net structure to form a new down-sampling network module called CSA. The proposed EMCA module is the core component of CSA, enhancing the depth and diversity of feature extraction through an improved multi-branch structure. This module offers a more comprehensive feature description and boosts the model’s generalization ability.
(3) We replaced the original YOLOv8 detection head with a new detection head module called DETECT_SPFPN. This change introduced a new contrast space generalized feature pyramid network (SPFPN) that enhances the concept of the feature pyramid network (FPN) for object detection. The SPFPN efficiently integrates multi-scale features, essential for capturing high-level semantics and low-level spatial details. To optimize performance under computing resources, feature maps of different scales utilize different channel dimensions.

## 2. Materials and Methods

### 2.1. Datasets

#### 2.1.1. Data Acquisition

The sample data were collected at Dong’Ao Deer Farm in Shuang Yang City, Jilin Province. The images were taken intermittently for a total of 10 days using a mobile phone. To enhance the dataset’s robustness, sika deer were photographed in various postures during different times of the day: morning, afternoon, evening, and during other routine activities. Image storage was done on an external portable hard drive, capturing a total of 596 sika deer images at a consistent resolution of 640 × 640 pixels. The remaining 579 sika deer sample data were sourced from the public animal four-category dataset on the Kaggle website [17], with the sika deer image resolution standardized to 640 × 640 pixels.

#### 2.1.2. Dataset Production

**Figure 1:**
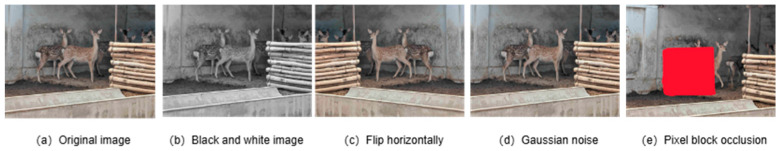
Images enhanced by different data augmentation method.

We utilized the lightweight graphic annotation software Labelimg to categorize sika deer into four postures: standing, lying, eating, and attacking. In this method, a total of 1175 sika deer image data were created and divided into an 8:2 ratio, resulting in a training set comprising 940 images and a validation set consisting of 235 images. To enhance the model’s generalization and robustness, and to prevent overfitting during training, this paper employed data augmentation techniques such as black and white images, Gaussian noise, random flipping, and adding pixel block occlusion. The image effects after data augmentation are illustrated in Figure 1. Following data augmentation, the training set expanded to 3760 images while the validation set remained at 940 images. The sika deer postures were categorized into four groups: standing, lying, eating, and attacking. Specific information is detailed in Table 1.

### 2.2. The Proposed Improved GFI-YOLOv8

**Figure 2:**
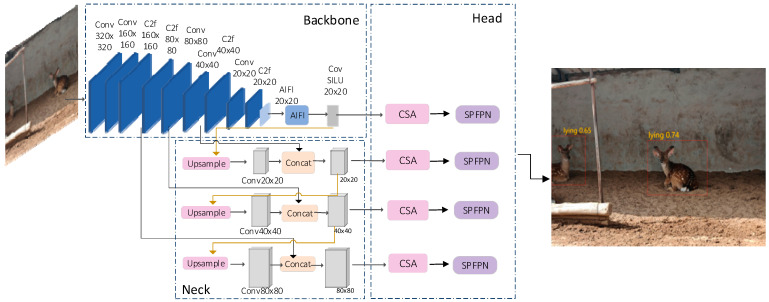
The overall structure of the GFI-YOLOv8 model.

YOLOv8 is the latest model in the YOLO series, based on the family of target detection models introduced by Ultralytics [18]. These models are cutting-edge SOTA (state-of-the-art) models in the field. In this paper, we propose an improved YOLOv8n-based model for target detection of sika deer—the GFI-YOLOv8 model. The backbone mainly consists of two modules, C2f_iAFF and AIFI, which extract features from the input image, respectively. The C2f_iAFF module acquires rich gradient information while ensuring lightweight design. The AIFI module utilizes the self-attention mechanism to process high-level features in the image. The neck uses the feature pyramid network and path aggregation network structure to enhance the feature fusion capability of the network by combining features of different scales. The CSA module replaces the C2f module in the head network, and the SPFPN module replaces the original pyramid network structure. The modified model structure is illustrated in Figure 2.

#### 2.2.1. Modification of Backbone Network Structure

**Figure 3:**
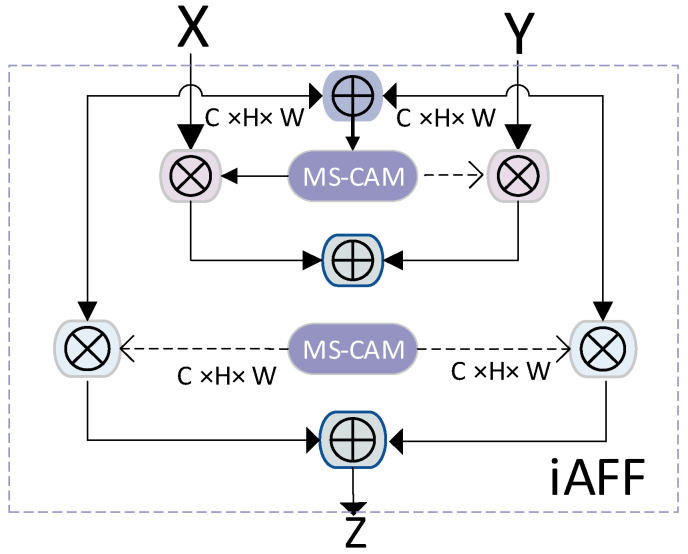
iAFF model structure diagram.

The bottleneck structure is commonly utilized in deep networks and was initially introduced in ResNet. The C2f class is a key component in the YOLO algorithm. This class is a neural network module inherited from nn.Module. In this article, we introduced the iAFF module from the Attentional Feature Fusion paper to enhance the performance of the sika deer posture recognition network. The structure of the iAFF module is illustrated in Figure 3. The iAFF model addresses the bottleneck that may arise from the initial integration of feature maps through iterative attention feature fusion. This approach enables the model to achieve superior results even with fewer layers or parameters.

**Figure 4:**
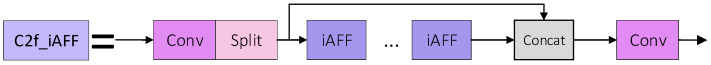
C2f_iAFF structure diagram.

The improved C2f-iAFF structure diagram is obtained, as shown in Figure 4. The enhanced C2f module conducts feature extraction and transformation on the input data through two convolutional layers (cv1 and cv2). It divides the input data into two branches, where one branch is directly sent to the output, while the other branch goes through multiple bottleneck modules and the iAFF module for processing. This process boosts the network’s nonlinear and representation capabilities. The C2f module achieves feature fusion by combining features from different branches in the channel dimension, enhancing the expressive power of features. These enhancements in the C2f-iAFF structure significantly improve the model’s feature fusion ability, leading to notable performance enhancements in the sika deer posture recognition task.

**Figure 5:**
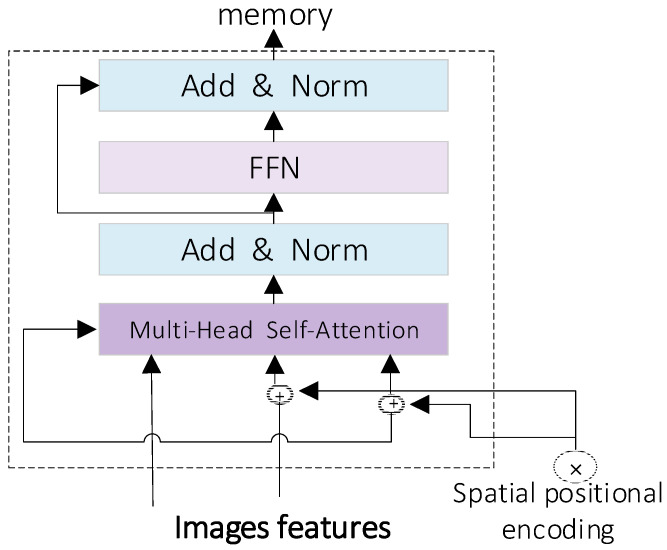
AIFI module structure diagram.

We utilize the attention-based intra-scale feature interaction module (AIFI) in the RT-DERT module to replace the original SPPF module. Large kernel convolutional neural networks have garnered significant attention in recent years. They increase the size of the convolution kernel to expand the receptive field, thereby enhancing the expressiveness of the model. However, large kernel convolution faces two primary challenges in practical applications: one is the substantial computational load, and the other is the potential introduction of redundant information. To address these issues, we incorporate the AIFI (attention-based intra-scale feature interaction) module in the RT-DETR model, which, along with the cross-scale feature fusion module (CCFM) based on CNN, forms the encoder section of the model. The core structure of the AIFI model is illustrated in the figure. The main model structure of AIFI is shown in Figure 5.

The AIFI module utilizes the self-attention mechanism to handle high-level features in the image. Self-attention is a mechanism that enables the model to consider other pertinent parts of the data while processing a specific part. This approach is especially apt for processing high-level image features with abundant semantic information. Recognizing that the high-level feature layer embodies richer semantic concepts, it can better capture the relationships between conceptual entities in the image. Simultaneously, it mitigates the deficiency of essential semantic depth in low-level features, which could result in data processing redundancy and ambiguity.

#### 2.2.2. Improved Convolutional Neural Network Module and Attention Mechanism Module

##### EMCA Module

**Figure 6:**
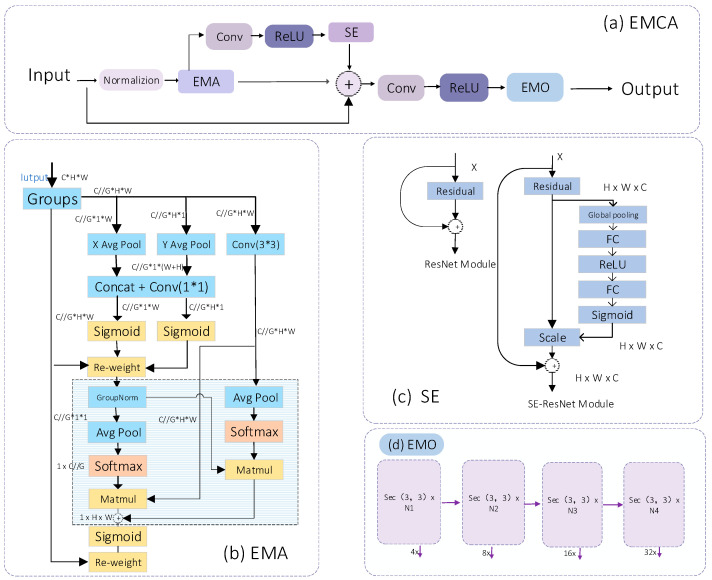
(**a**) is the overall structure module of EMCA, (**b**) is a structural diagram of an efficient multi-scale attention module for cross-spatial learning, (**c**) is the channel attention mechanism module, and (**d**) is the downstream task of the reverse residual struct.

We have developed a new, efficient multi-scale fusion attention mechanism that combines the spatial attention branch with the channel attention branch. The input data are standardized and processed through this mechanism, followed by passing them through the convolution layer and activation function using the channel attention mechanism. Downsampling is employed to emphasize or diminish features while maintaining high resolution within the module to mitigate the potential loss of high-resolution information in deep neural networks. We have designed EMCA to fully collapse the feature layer in one dimension, and, ultimately, convert the integrated data into the required output model size through the convolution layer and activation function in the same dimension. Its structure is shown in Figure 6.

In contrast, the multi-scale spatial attention mechanism in universities requires the input data to pass through two distinct branches via the pooling layer. Subsequently, the data go through upsampling and convolution layers multiple times to acquire the necessary weights and output results. This process increases the data volume for the weights and output results. Consequently, a channel attention mechanism is incorporated, and a reverse residual block is added to transmit the output result back to the 1 × 1 convolution layer, thereby reducing the data volume.

**Figure 7:**
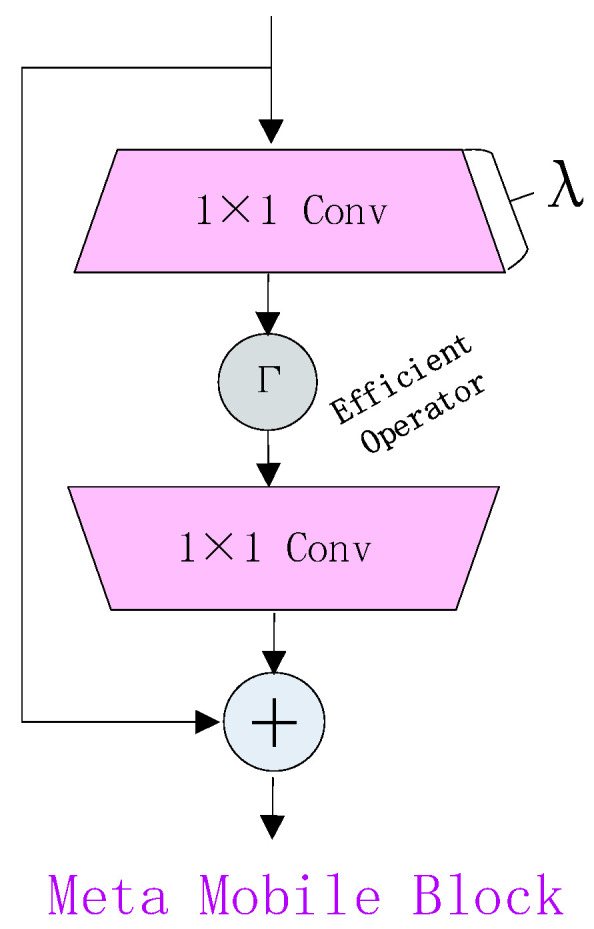
MMB structure diagram.

We rethink Inverted Residual Block in MobileNetv2 [19] with core modules in Transformer [20], and inductively abstract a general Meta Mobile Block (MMB) in Figure 7, which takes parametric arguments expansion ratio λ and efficient operator F to instantiate different modules.

Taking the image input $Χ\left(Χ\in RC\times H\times W\right)$ as an example, MMB first expands the channel dimension using the extended MLP with an output/input ratio equal to *λ*.

(1) $$X_{e}=MLP_{e}\left(X\right)\left(\in R^{\lambda C\times H\times W}\right)$$

Finally, a shrinking MLP with an inverse input-to-output ratio equal to *λ* is used to reduce the channel size.

(2) $$X_{f}=\Gamma P_{e}\left(X\right)\left(\in R^{\lambda C\times H\times W}\right)$$

The residual connection is used to obtain the final output. ${Y=X+X}_{f}\left(\in R^{\lambda C\times H\times W}\right)$ Normalization and activation functions are omitted for clarity.

EMCA maintains the highest resolution within the module in both channel and spatial dimensions. In the multi-scale fusion attention mechanism, a reverse residual is added to the last input feature layer at the university to create an efficient model akin to ResNet [21] for downstream tasks, thereby reducing data transmission time.

##### CSA Module

**Figure 8:**
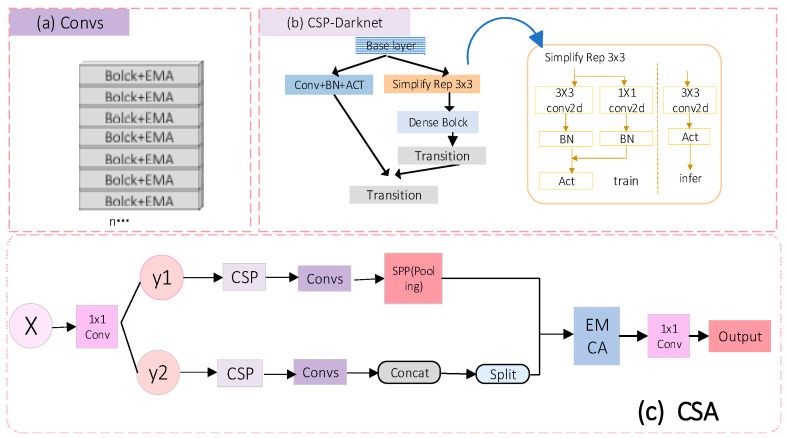
(**a**) is the convolutional block structure, (**b**) is the CSP-Darknet module structure, and (**c**) is the CSA module structure.

Convolutional neural networks have enabled state-of-the-art methods to achieve incredible results on computer vision tasks such as object detection. In this paper, we introduce the Cross Stage Partial Network (CSP-Net) to alleviate the problem of previous works requiring large amounts of inference computation from a network architecture perspective [22]. CSP-Darknet is a convolutional neural network and object detection backbone developed in 202022. The architecture uses a series of CSP blocks with an increasing number of layers; the output of each block is concatenated with the output of the corresponding block in the previous stage. This allows the network to learn both fine and coarse features of the input. The final output is obtained by applying a convolutional layer to the feature map generated by the last CSP block. Based on this, we propose a new convolutional neural network module (CSA), as shown in Figure 8.

The CSA module inherits and develops the Cross Stage Partial Network design concept of CSP-Net, achieving enhanced sensitivity to gradient changes by integrating feature maps in multiple stages of the network. Optimizing the flow of gradients also helps maintain the richness and diversity of information in the deep layers of the network, significantly improving the efficiency and accuracy of feature extraction. During data transmission, the input features are divided into two channels, namely, y1 and y2. Each residual block in the network is followed by an EMA module [23]. The number of channels is then restored to the original data throughput through SPP, up-sampling, and down-sampling. The final merging branch passes the input features through an EMCA module and then outputs the obtained feature data through a 1 × 1 convolution. The EMA module enhances the smoothness of feature representation by applying exponentially weighted averaging on feature maps, reducing noise, and retaining important feature information. The EMCA module is the core part of CSA, further enhancing the depth and diversity of feature extraction through an improved multi-branch structure. CSA can effectively capture key information in the image, achieving more accurate feature representation of small texture features or the overall image structure. Additionally, this cross-stage feature integration helps reduce overfitting during network training as feature maps at different stages complement each other, providing a more comprehensive feature description and enhancing the model’s generalization ability.

#### 2.2.3. Improved Detection Head Derect Module: SPFPN

**Figure 9:**
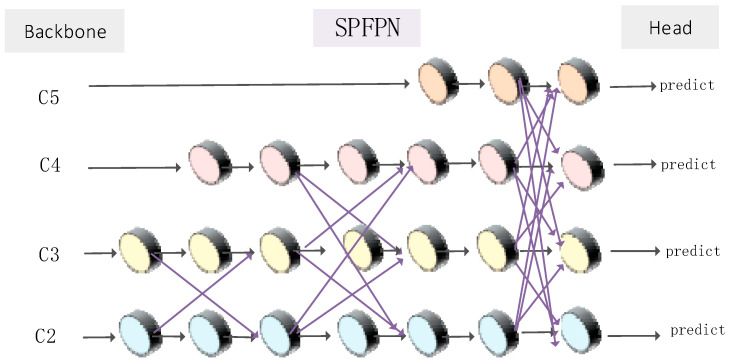
Structural diagram of SPFPN module.

In order to enhance the accuracy of sika deer posture recognition, we have introduced an improved feature pyramid network module named Spatially Aware Feature Pyramid Network (SPFPN). Unlike the traditional FPN [24,25,26], SPFPN adjusts the weights of feature maps of various scales adaptively by incorporating a high-level multi-scale fusion attention mechanism, enabling a more efficient capture of the spatial information of the target. SPFPN comprises multiple parallel feature extraction layers, each designed to enhance feature representation through a self-focused module. It employs strategic up-sampling and down-sampling techniques to facilitate the efficient fusion of features across various scales while preserving high resolution. Additionally, the concept of asymptotic fusion is implemented to ensure that features from different levels progressively converge during the fusion process, thereby minimizing the semantic gap between them. The proposed SPFPN architecture is shown in Figure 9.

Because the feature scales corresponding to the feature maps within the same feature layer exhibit a significant semantic gap—particularly due to the differing weights of parallel scale feature maps—direct fusion can result in a substantial semantic disparity. To address this issue, we have designed the structure of the Scale-Pyramid Feature Pyramid Network (SPFPN) to be asymptotic and have integrated advanced multi-location fusion attention mechanisms. This allows for the fusion of parallel feature mappings, enabling feature transmission to achieve cross-space interaction. Consequently, this structure facilitates a closer integration of semantic features across different levels.

## 3. Experimental Results and Analysis

### 3.1. Evaluation Indicators

We evaluated the performance on the sika deer test images using precision, recall, AP50, mAP50, number of parameters, and GFLOPs. In object detection, *TP* represents the number of correctly identified object boxes, *FP* represents the number of incorrectly identified object boxes, and *FN* is the number of missed object boxes, calculated by subtracting *TP* from the total number of object boxes. The default value of mAP50 is set to mAP50-95, representing the average mAP50, with a step increment of 0.05. The calculation formula is as follows:

(3) $$P=\frac{TP}{TP+FP}$$

(4) $$R=\frac{TP}{TP+FN}$$

(5) $$AP=\int_{0}^{1}P(r)dr$$

(6) $$mAP=\frac{\sum_{I=1}^{N}AP_{i}}{N}$$

**Figure 10:**
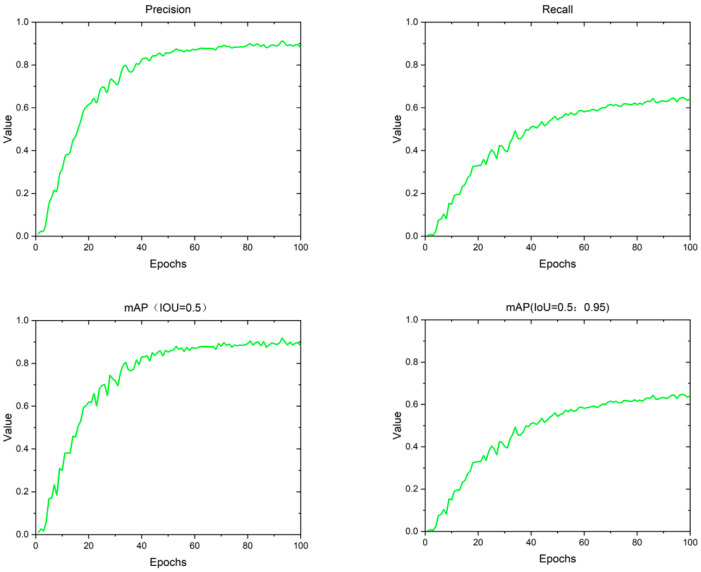
Curve graph of various evaluation indicators changing with the number of training times.

According to the log file, we can plot the curve depicting changes in the evaluation indices as the number of training epochs increases during the training process, as illustrated in Figure 10. The graph below displays the accuracy (Precision), recall (Recall), mAP50, and mAP50-95 of the GFI-YOLOv8 model utilized for sika deer posture recognition detection over 100 training rounds. The value of each evaluation index gradually stabilizes starting from the 95th round.

### 3.2. Experimental Details

This study uses the same equipment for experiments, and the model is based on the PyTorch deep learning framework and developed in the Anaconda environment. Table 2 shows the main experimental equipment environment configuration. The experimental hyperparameters are set as follows: the number of iterations is 100, the batch size is 32, the optimizer is SGD, the initial learning rate is 0.01, the learning rate momentum is 0.937, the weight attenuation coefficient is 0.0005, and the model is trained using one GPU.

#### 3.2.1. Comparative Experiments of Different Attention Mechanisms

This paper utilizes the YOLOv8n network as the foundational model and evaluates its performance using accuracy (Precision), recall rate (Recall), average precision (mAP), parameter quantity (Params), and floating-point operation number (GFLOPs) as key indicators. The study compares the performance of YOLOv8n, YOLOv8n-iRMB [27], YOLOv8n-EMA, YOLOv8-SE [28], and YOLOv8n-EMCA. The experimental results are shown in Table 3. After comparison, the EMCA structure is obviously superior to the other three attention mechanisms in precision, recall rate, and mAP50-95. The EMCA module demonstrates superior results in this experiment.

#### 3.2.2. Comparison of Different Feature Extraction Backbone Networks in Head Networks

In order to evaluate the performance of the best head backbone network, CSA is compared with YOLOv8, YOLOv8_CSPS, CSPS_EMA, and CSPS_iRMB. The experimental results are shown in Table 4. It shows that CSA is significantly higher than other modules in both precision and recall. CSA only achieved 86.5% accuracy in mAP50, which did not exceed the other three modules; however, its mAP50-95 was higher than the other four modules. Therefore, the CSA module was selected to enhance the structure of the head network.

#### 3.2.3. Comparison of Different Improved C2f Networks

In order to compare the experimental results of the improved C2f module, we conducted the following comparative experiments. We added EMA, iRMB, AKConv [29], Faster_block, EMCA, and iAFF modules, respectively. The experimental results are shown in Table 5. It shows that C2f-iAFF structure has the highest mAP50-95, Recall (R), and mAP50. The improved structures based on the C2f-iAFF framework also demonstrate good performance.

### 3.3. Ablation Experiment

Through the experimental comparison of different modules, it is concluded that this study introduces an iterative attention mechanism in the C2f module to form a new C2f_iAFF module based on YOLOv8n. It replaces the original SPPF module with an attention-based internal scale feature interaction module (AIFI). Simultaneously, a new convolutional neural network structure (CSA) is proposed, and the original detection head is replaced by the SPFPN module. Finally, the feature extraction ability and reasoning speed of the model are enhanced. To study the various improved modules introduced in the model recognition results, the variable control method is used to design ablation experiments to obtain different improved models. Then, the improved model is trained using the same training method. The experimental results are shown in Table 6. It can be intuitively seen from the table that in addition to the recall rate, our improved module has the best effect in terms of precision, mAP50, and mAP50-95.

#### Comparative Experiments on the Performance of Different Network Models

In order to evaluate the posture recognition capabilities of the enhanced model presented in this paper, we compared it with current mainstream detection models under identical conditions: YOLOv5n, YOLOv7-tiny, YOLOv8n, YOLOv8, YOLOv9, and YOLOv10 benchmark models. Detailed experimental results are provided in Table 7. As indicated in Table 7, the GFI-YOLOv8 model proposed in this study was assessed using self-constructed datasets. In terms of precision (P), mean average precision (mAP), and other evaluation metrics, it outperforms the mainstream models YOLOv5, YOLOv7-tiny [30], YOLOv8n, YOLOv8, YOLOv9 [31], and YOLOv10 [32], although it falls short of YOLOv9 in recall rate. However, precision and parameter count are superior to those of YOLOv9. Despite the incorporation of new modules, the model remains competitive with existing models regarding parameter count (Params) and floating-point operations (GFLOPs), demonstrating superior performance and generalization compared to all models except YOLOv10, YOLOv5n, and YOLOv8n. Under the same experimental conditions, the enhanced algorithm proposed in this study achieves higher recognition accuracy for sika deer posture recognition, with the mAP50 and mAP50-95 values also being the highest. This study demonstrates that the enhanced algorithm model exhibits excellent adaptability while maintaining high accuracy, particularly in meeting the requirements of sika deer breeding.

**Figure 11:**
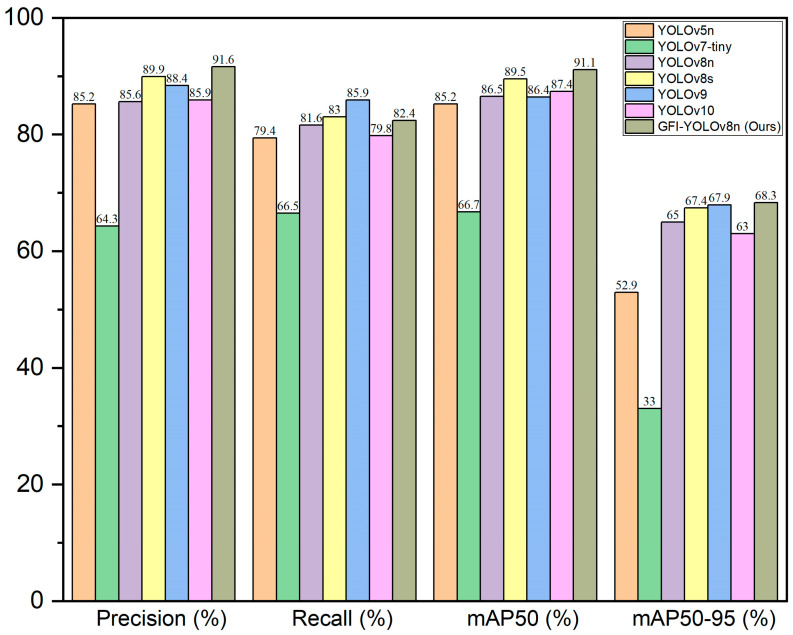
Performance comparison of 7 detection algorithms.

**Figure 12:**
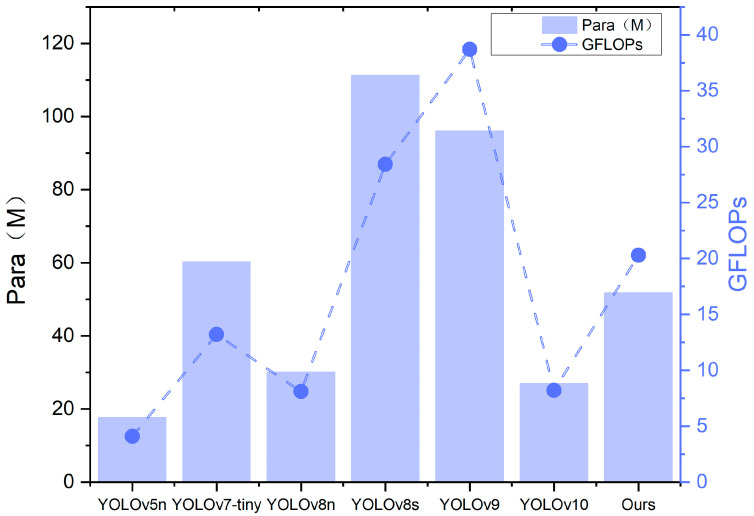
Comparison of the computational volume of the 7 models.

**Figure 13:**
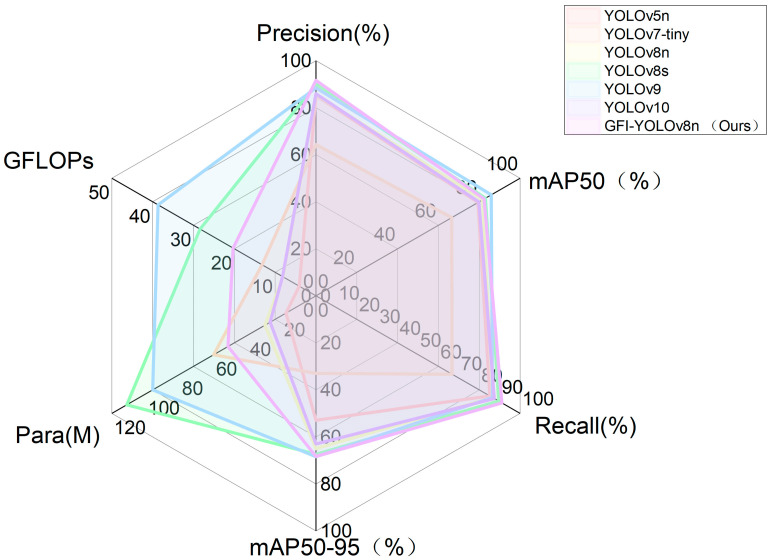
Comparison of the comprehensive performance of 7 models.

In order to more intuitively demonstrate the advantages of this model, we evaluate the precision (P), recall (R), mAP50 (%) and mAP50-95 (%), model size (Params), and floating-point operations (GFLOPs). The performance comparison of seven different algorithms is shown in Figure 11, Figure 12 and Figure 13.

### 3.4. Heat Map Visualization Analysis

**Figure 14:**
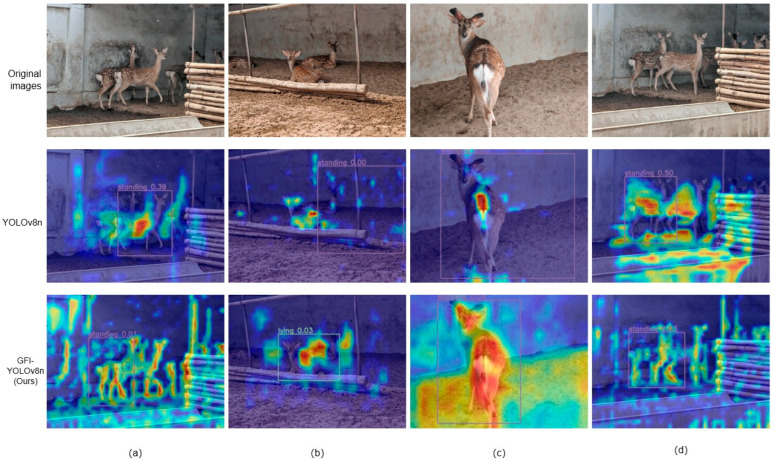
Comparison of heat maps before and after model optimization. A darker color means a larger data value, and a lighter color means a smaller data value. (**a**–**d**) are images with different poses and poses, respectively.

We have better demonstrated the better adaptability of this model through some visual analysis. The figure below is a comparison of YOLOv8n and my model (GFI-YOLOv8) using heat maps. Heat maps usually use gradient colors to represent the size of data values [33]. The color changes from light to dark, or from one color to another, to show the low value to high value of the data. The change in color can be likened to the change in temperature, where colder colors (such as blue or green) represent lower values and warmer colors (such as red or yellow) represent higher values. As shown in Figure 14, it can be seen that the heat map image displayed by our model is more effective, which can better cover the recognized graphics, and the depth of the color can represent the confidence of the recognition.

## 4. Discussion

In this study, GFI-YOLOv8 has effectively addressed the previous research gap in the field of long-distance, non-contact attitude recognition of sika deer. Research on sika deer pose recognition provides breeders with a valuable tool to accurately assess their breeding status and enables comprehensive monitoring of changes in sika deer poses over time. With this technology, farmers can analyze the breeding status of sika deer with greater precision and monitor their behavior and health in real time, thereby optimizing breeding management. This demonstrates that our approach has significant potential for widespread application and value.

The design concept of the GFI-YOLOv8 model is mainly reflected in three aspects. Firstly, we introduce C2f_iAFF and AIFI models to enhance the integration ability of multi-scale features. These models use an iterative attention mechanism to continuously process data features at different levels to improve the fusion effect of features at different scales. This design effectively solves the defects of the original model in extracting target edge and texture information, thus improving the accuracy of target detection. Secondly, to enhance the depth and diversity of feature extraction, we developed the CSA module. This module is based on the Convolutional Neural Network (CSP) and EMCA module as the core. The EMCA module divides the input feature map into two parts and uses an efficient multi-scale fusion attention mechanism to deeply analyze the interrelationships between different features. This spatial analysis captures the relative position relationship between the global context information and the feature map effectively. Finally, to integrate features from different modules more efficiently, we designed a new detection head module. By integrating an efficient multi-scale fusion attention mechanism, SPFPN can adjust the weights of different scale feature maps adaptively, capturing the spatial information of the target more accurately. This method avoids the information redundancy that may be caused by simple feature concatenation in the original model, further improving the overall performance of the model.

Although the model proposed in this paper achieves accuracy and speed in deer target detection, it also has limitations. Due to the computing requirements of the kernel, the size of the model increases, especially in resource-constrained environments, which may make it difficult to adapt the model to all server devices. When the model processes deer images with severe occlusion, the feature information is lost due to occlusion, or the model’s generalization ability is insufficient. In future research, we will strive to enhance the accuracy of occluded object detection. Simultaneously, we will optimize the model structure, and reduce the calculation amount and model size to improve the adaptability and deployment efficiency of the model on various devices. In the future, we will explore the model’s performance in more complex environments, enhance its robustness and predictability, and integrate it with other deep learning techniques and optimization methods to further reduce the total cost of model computation and enhance real-time processing power.

## 5. Conclusions

This paper proposes an enhanced model GFI-YOLOv8 based on YOLOv8, specifically optimized for sika deer posture recognition. The paper suggests the C2f module should be replaced with the C2f_iAFF module. The module’s capacity for data generalization has been strengthened, leading to an improvement in accuracy rates. The AIFI module replaces the SPPF module and focuses on processing high-level image features through a self-attention mechanism, which enhances the detection of high-level image features. The designed CSA module enhances the depth and diversity of feature extraction through the reverse residual structure of the core part of EMCA, addressing the issue of overly large models caused by multi-level data input. The original YOLOv8 detection head is replaced with the DETECT-SAFPN detection head, and the SPFPN optimized feature pyramid network is introduced. By expanding the data input method from single-branch to multi-branch input, the receptive field is increased, enhancing the accuracy of sika deer posture recognition target detection. While enhancing the accuracy of sika deer posture recognition, GFI-YOLOv8 also maintains high detection speed and low computational cost. The experimental results demonstrate that GFI-YOLOv8 performs well on the self-built sika deer dataset, achieving an accuracy of 91.6% and a mAP50 improvement of 4.6%. Compared to other models in the YOLO series, it offers comprehensive performance advantages in terms of parameter volume, GFLOPs, detection speed, and accuracy. Our work provides a meaningful exploration for the accurate identification of sika deer poses. In our future work, we aim to further enhance dataset richness, model performance, and system functionality.

## Figures and Tables

**Table 1:** Definition of sika deer posture classification.

Posture Category	The Name of the Pose	Pose Definition
Standing	Standing	Sika deer have at least three legs standing on the ground at the same time
Lying	Lying	The abdomen of the body is in contact with the ground, and the hooves and legs are not used to support the body
Eating	Eating	The mouth of the sika deer touches the ground, or the sika deer is standing next to the trough, or the small sika deer is feeding on its mother’s milk
Attacking	Bumping, kicking, Chasing	The act of striking another deer’s body or antlers with its horns, sika deer kicking or kicking the other body with its front foot, or before or after a fight

**Table 2:** Experimental environment configuration.

Environment Configuration	Parameter
Operating system	Windows10
CPU	Intel(R) Core(TM) i9-10920X CPU @ 3.50GHz
GPU	NVIDIA GeForce RTX 3080
Development environment	PyCharm 2023.2.5
Language	Python 3.9.6
Framework	PyTorch 2.0.1
Operating platform	CUDA 11.8

**Table 3:** Comparison of different attention mechanisms.

Methods	Precision (%)	Recall (%)	mAP50 (%)	mAP50-95 (%)	Para (M)	GFLOPs
YOLOv8n	85.6	81.6	85.6	65	30.06	8.1
EMA	90.5	80.4	89.2	66.8	30.06	8.1
iRMB	90.3	85.2	90.1	67	33.56	8.3
SE	88.7	83.4	86.4	67.8	31.45	8.3
EMCA	91.3	85.4	84.6	68.8	30.51	8.4

**Table 4:** Comparison of different feature extraction backbone networks in head networks.

Methods	Precision (%)	Recall (%)	mAP50 (%)	mAP50-95(%)	Para (M)	GFLOPs
YOLOv8n	85.6	81.6	85.6	65	30.06	8.1
YOLOv8n + CSPS	90.1	84.1	90.3	67.8	29.83	8.1
CSPS + EMA	89.3	84.2	89.6	67.3	29.91	8.2
CSPS + iRMB	85.1	84.6	88.5	67.4	32.79	8.9
CSA	90.3	86.4	86.5	68.5	33.54	8.1

**Table 5:** Comparison of different improved C2f networks.

Methods	Precision (%)	Recall (%)	mAP50 (%)	mAP50-95 (%)	Para (M)	GFLOPs
C2f_EMA	87.3	81.8	86.3	62.7	29.91	8.1
C2f_iRMB	84.2	83.1	86.3	63.6	29.83	8.1
C2f_AKConv	86.1	80.4	85.6	65.4	23.67	8.2
C2f_Faster	83.5	81.9	86.8	62.7	32.79	8.0
C2f_EMCA	87.8	81.7	87.5	64.7	33.54	8.1
C2f_iAFF	88.7	83.4	89.8	66.2	30.52	8.1

**Table 6:** Ablation experiment.

NO.	C2f_iAFF	AIFI	CSA	SPFPN	Precision (%)	Recall (%)	mAP50 (%)	mAP50-95 (%)	Para (M)	GFLOPs
1	√				88.7	83.4	89.8	66.2	30.52	8.1
2		√			89.1	83.4	89.6	67.6	31.2	8.4
3			√		90.3	86.4	86.5	68.5	33.54	8.1
4				√	87.6	82.6	83.1	65.1	8.8	30.5
5	√	√			88.4	83.7	84.7	64.8	8.4	29.7
6	√	√	√		89.8	83.4	88.5	66.1	8.9	32.7
7	√		√	√	90.9	84.0	87.4	65.6	19.6	48.8
8	√	√	√	√	91.6	82.4	91.1	68.3	51.74	20.3

**Table 7:** Comparison results of different network models for deer posture detection.

Methods	Precision (%)	Recall (%)	mAP50 (%)	mAP50-95 (%)	Para (M)	GFLOPs
YOLOv5n	85.2	79.4	85.2	52.9	17.64	4.1
YOLOv7-tiny	64.3	66.5	66.7	33	60.22	13.2
YOLOv8n	85.6	81.6	86.5	65	30.06	8.1
YOLOv8s	89.9	83	89.5	67.4	111.27	28.4
YOLOv9	88.4	85.9	86.4	67.9	96.01	38.7
YOLOv10	85.9	79.8	87.4	63	26.95	8.2
GFI-YOLOv8n (Ours)	91.6	82.4	91.1	68.3	51.74	20.3

## Data Availability

Data available on request due to restrictions.
